# Artificial TALE as a Convenient Protein Platform for Engineering Broad-Spectrum Resistance to Begomoviruses

**DOI:** 10.3390/v7082843

**Published:** 2015-08-20

**Authors:** Xiaofei Cheng, Fangfang Li, Jianyu Cai, Wei Chen, Nan Zhao, Yuqiang Sun, Yushuang Guo, Xiuling Yang, Xiaoyun Wu

**Affiliations:** 1 College of Life and Environmental Science, Hangzhou Normal University, Hangzhou 310036, Zhejiang, China; E-Mails: idave.01@163.com (W.C.); sunyuq1109@hotmail.com (Y.S.); 2 Institute of Biotechnology, Zhejiang University, Hangzhou 310029, Zhejiang, China; E-Mails: elva1988@163.com (F.L.); zhaonanzilv@163.com (N.Z.); xiulingyang@zju.edu.cn (X.Y.); 3 College of Agricultural and Food Science, Zhejiang Agricultural and Forestry University, Lin’an 311300, Zhejiang, China; E-Mail: cjy19920514@hotmail.com; 4 Key Laboratory of Molecular Genetics, China National Tobacco Corporation, Guizhou, Institute of Tobacco Science, Guiyang 550083, Guizhou, China; E-Mail: yshguo@126.com

**Keywords:** Antivirus, Begomovirus, Broad-spectrum resistance, DNA-binding protein, Transcription activator–like effectors

## Abstract

Transcription activator–like effectors (TALEs) are a class of sequence-specific DNA-binding proteins that utilize a simple and predictable modality to recognize target DNA. This unique characteristic allows for the rapid assembly of artificial TALEs, with high DNA binding specificity, to any target DNA sequences for the creation of customizable sequence-specific nucleases used in genome engineering. Here, we report the use of an artificial TALE protein as a convenient platform for designing broad-spectrum resistance to begomoviruses, one of the most destructive plant virus groups, which cause tremendous losses worldwide. We showed that artificial TALEs, which were assembled based on conserved sequence motifs within begomovirus genomes, could confer partial resistance in transgenic *Nicotiana benthamiana* to all three begomoviruses tested. Furthermore, the resistance was maintained even in the presence of their betasatellite. These results shed new light on the development of broad-spectrum resistance against DNA viruses, such as begomoviruses.

## 1. Introduction

The family *Geminiviridae* consists of a group of plant viruses with genomes comprised of circular, single-stranded DNA (ssDNAs). Viruses in this family cause severe disease in a broad range of crops worldwide [1]. Geminiviruses are classified into seven genera (*Mastrevirus*, *Curtovirus*, *Begomovirus*, *Topocuvirus*, *Eragrovirus*, *Turncurtovirus*, and *Becurtovirus*) which are divided based on genome organization, host range, vector specificity, and phylogenetic relationship [2]. *Begomovirus* is the largest genus of the seven genera which not only contains most of geminiviruses identified thus far (288 out of 325), but also includes the most economically important geminiviruses [3]. The genome of begomoviruses consists of one (monopartite) or two (bipartite) molecules of circular ssDNAs of approximately 2.6–2.8 kb in length [4]. Furthermore, begomoviruses are often found associated with satellites, including alphasatellite and betasatellite. These satellites are dependent on helper viruses for their replication, encapsidation, and transmission between hosts. However, satellites may benefit the helper virus in many aspects, such as suppressing RNA silencing [5] and the suppression of jasmonic acid defense in plants [6]. Rapid evolution through mutation, recombination, and pseudorecombination makes begomoviruses one of the most destructive plant viruses in global agriculture [7,8].

To counter begomovirus disease researchers have tried many strategies, including conventional crop breeding, transgenic approaches by expressing viral proteins or non-pathogen derived antiviral agents, and RNA silencing [9,10,11]. Although several field trials have been conducted, commercially-available resistant crops, especially those with broad-spectrum resistance, are rare. Expression of DNA-binding proteins in transgenic plants, *i.e.*, zinc-finger proteins, that specifically bind to begomovirus genomic DNA, can confer tolerance to the infection with corresponding begomovirus [12,13]. In our previous study, we showed that rationally-designed artificial zinc-finger nucleases, which recognize a conserved sequence motif within begomovirus genomes, can inhibit the replication of multiple begomoviruses [14]. These studies suggested that artificial DNA binding protein techniques may be a promising method for developing broad-spectrum resistance to begomoviruses.

Transcription activator–like effectors (TALEs) are a family of virulence factors encoded by the *Xanthomonas* genus of plant pathogens. These proteins are injected into the host cell to modulate the expression of host genes by directly binding to promoter sequences. Basically, TALEs can be subdivided into three domains: N-terminal type III secretion signaling domain, central DNA binding domain, and C-terminal nuclear localization and activation domain. The DNA binding domain naturally consists of 17–18 repeats of highly conserved 33–35 amino acid motif that are arranged side by side. The amino acids at positions 12 and 13 within each repeat, known as variant di-residue (RVD), determine its DNA binding specificity. For example, the naturally-occurring RVD of asparagine-isoleucine (NI) recognizes adenine (A), while histidine-aspartic acid (HD) recognizes cytosine (C), asparagine-glycine (NG) recognizes thymine (T), and asparagine-asparagine (NN) recognizes guanine (G) or A [15,16]. This simple and unique DNA recognition system of TALE effectors allows for the quick assembly of artificial TALE effectors with high DNA binding specificity to any target DNA sequences. Consequently, TALE has been rapidly and widely exploited in biotechnology to design and build customizable transcription factors and sequence-specific nucleases for genome editing [17]. Compared to artificial zinc-finger technology, construction of artificial TALEs with high affinity and specificity is less time consuming and is more cost effective [18]. These advantages prompted us to test whether it can be used as a convenient platform for developing broad-spectrum resistance to DNA viruses, *i.e.*, begomoviruses.

## 2. Results and Discussion

**Figure 1:**
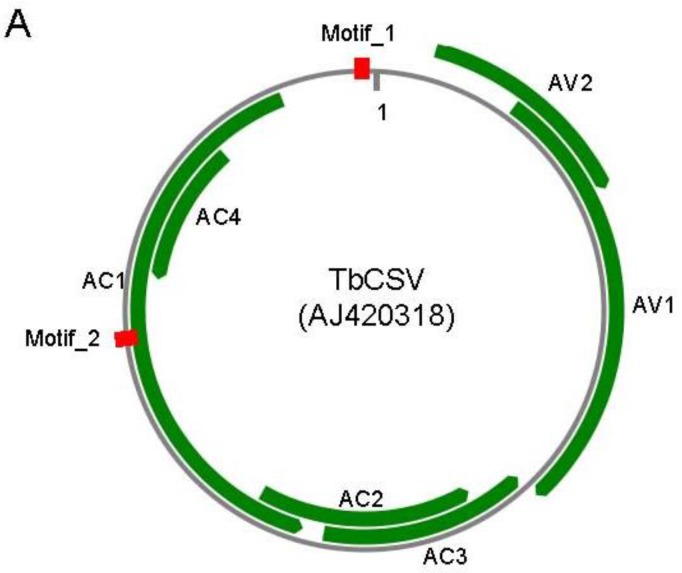

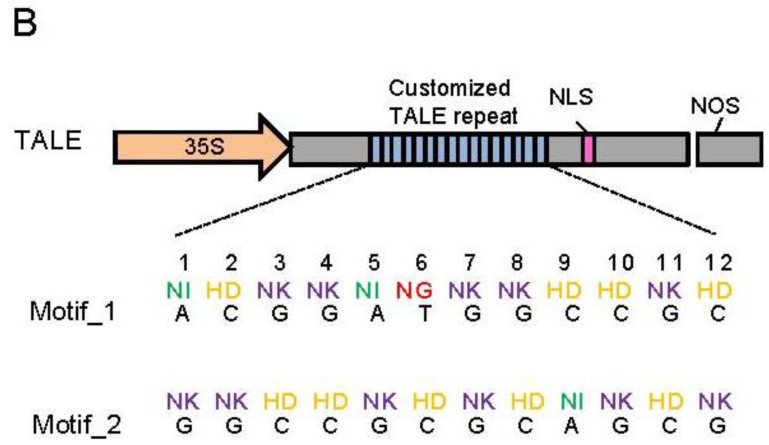
Diagram illustrates of target sequences and artificial TALEs. (**A**) Locations of target sequences in TbCSV genome. TbCSV genome is shown as black circle, and TbCSV encoded genes are shown as green arrows. The two conserved sequence motifs (Motif_1 and Motif_2) were highlighted in red. AC1, replication-associated protein (Rep); AC2, transcriptional activator protein (TrAP); AC3, replication enhancer protein (REn); AC4, RNA-silencing suppressor; AV1, coat protein (CP); AV2, precoat protein; (**B**) Schematic representation of artificial TALE proteins. RVDs in the tandem repeats and their corresponding 12-bp DNA-binding targets were enlarged at the bottom. 35S, 35S promoter. NOS, NOS terminator. NLS, nuclear localization signal. Note that the TALE protein is not drawn in scale.

To find an artificial TALE target, we aligned all the genome sequences of monopartite begomoviruses available in GenBank (*n* = 288) to obtain the most conserved sequence motif. Initially, we tried to find conserved motifs which are long enough for constructing TALE-based nucleases (TALEN) to directly cleave the begomovirus genome [19]. However, begomoviruses are not highly conserved and it was impossible to find such a sequence motif. As an alternative, we tried to find the most conserved sequence motif in these begomovirus genomes with a length longer than 12 nt to ensure DNA binding specificity using an in-house BioPython script (available upon request). Candidates were searched against the *Nicotiana benthamiana* genome and transcript database to avoid possible off-target binding effects. Those candidates with complementary sequences to *N. benthamiana* genomic sequences or transcripts were discarded. Finally, two 12-nt motifs, motif_1 (5′-ACGGATGGCCGC-3′) and motif_2 (5′-GGCCGCGCAGCG-3′), which localize at the conserved hairpin and AC1 ORF of Tobacco curly shoot virus (TbCSV, accession number: AJ420318), respectively (Figure 1A), were utilized in this study. Motif_1 was conserved in all begomoviruses analyzed, whereas motif_2 was conserved in 98% of begomoviruses analyzed.

To construct artificial TALEs that can bind to the two candidate motifs, we synthesized four double-repeat units that recognize the dinucleotides AA, TT, GG and CC, respectively (Supplementary Text 1). Within these double-repeat units, the A, C, and T were recognized by naturally-occurring RVD NI, HD, and NG, respectively [15,16]. Whereas, the G was designed to be recognized by an artificial RVD: asparagine-lysine (NK), which was reported to have a higher specificity to G [20,21]. By using overlapping polymerase chain reaction (PCR), we then obtained all 16 dual-TALE repeats, with each recognizing one of the 16 dinucleotides (Supplementary Text 1). These units were assembled into artificial TALEs based on target sequences using a method called unit assembly, which involves the isocaudomer restriction enzymes, *Nhe*I and *Spe*I, to assemble the tandem repeats [22]. After assembly, the TALE was excised by *Spe*I and *Nhe*I double-digestion and then inserted into the unique *Nhe*I restriction site of Flank_seq1 to construct the full-length artificial TALE proteins (Supplementary Text 1). Flank_seq1 consists of the partial N- terminal domain, a half TALE repeat unit, and the nuclear localization sequences of a naturally occurring TALE effector. Finally, two artificial TALE genes (*TALE1* and *TALE2*) which encode proteins that can specifically recognize motif_1 and motif_2, respectively, were generated (Figure 1B and Supplementary Text 1). The DNA binding abilities of TALE1 and TALE2 protein to their target sequences were determined by electrophoretic mobility shift assay (EMSA) with recombinant TALE1 and TALE2 proteins (data not shown). These two TALE genes were then inserted into the p2300-35S vector between *Kpn*I and *BamH*I restriction sites to construct p2300-35S:TALE1 and p2300-35S:TALE2, respectively. These two plasmids were transformed into *Agrobacterium tumefaciens* strain GV3101 via electroporation. Transgenic *N. benthamiana* plants were obtained through leaf disc transformation, as reported earlier [23]. PCR was performed with primer 35S-F (5′-ACATGGTGGAGCACGACACG-3′) and 35S-R (5′-GAGGAAGGGTCTTGCGAAGG-3′) to identify plants with the T-DNA insertion. Reverse transcription-PCR (RT-PCR) was performed with primers TALE-F (5′-GTGGATCTACGCACGCTCGGCTAC-3′) and TALE-R (5′-GATGGCCACCACCTGGTCCGGGG-3′) to confirm the expression of *TALE1* and *TALE2* in transgenic plants. A 270 bp fragment of *N. benthamiana glyderaldehyde-3-phosphate dehydrogenase (GAPDH)* was amplified with primers GAPDH-F (5′-GCAGTGAACGACCCATTTATCTC-3′) and GAPDH-R (5′-AACCTTCTTGGCACCACCCT-3′) as an internal control. We identified five independent transgenic lines that express TALE1 and three that expressed TALE2 (Supplementary Figure 1). These plants did not have any obvious phenotypic differences and had similar growth rates when compared to wild-type plants (Supplementary Figure 1), suggesting that the expression of TALE1 and TALE2 had no obvious influence on normal *N. benthamiana* development.

**Figure 2:**
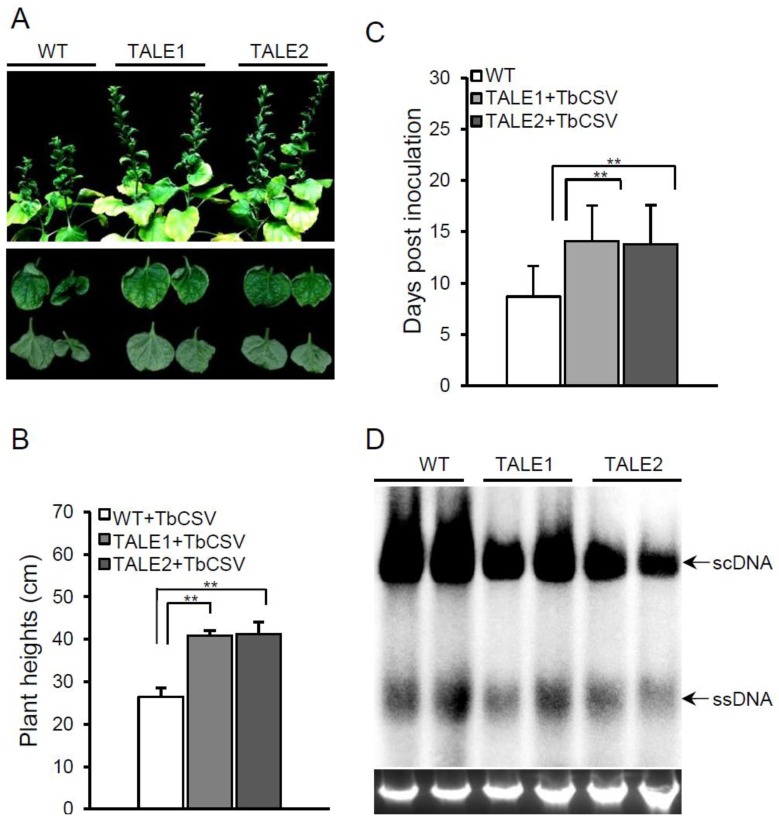
Transgenic plants show resistance to TbCSV infection. (**A**) The symptoms induced by TbCSV on wild-type, TALE1, and TALE2 transgenic *N. benthamiana* plants. A detailed view of virus symptoms on leaves was shown at the bottom panel. Pictures were taken at 20 dpi; (**B**) Average plant height of wild-type, TALE1, and TALE2 transgenic *N. benthamiana* plants infected by TbCSV at 20 dpi. Numbers were calculated from two independent trials with each trial of 10 plants. ** indicates *p <* 0.01; (**C**) Average time for TbCSV inducing viral symptoms on systemic leaves of wild-type, TALE1, and TALE2 transgenic plants. Numbers were calculated from two independent trials with each trial of 10 plants. ** indicates *p <* 0.01; (**D**) Southern blot detection of TbCSV genomic DNA in wild-type, TALE1, and TALE2 transgenic plants. An ethidium bromide stained gel was shown at the bottom for indicating DNA loadings. scDNA, supercoiled DNA; ssDNA, single-stranded DNA.

To determine whether the artificial TALEs can confer viral resistance against the infection of begomoviruses, we challenged TALE1 and TALE2 transgenic lines with TbCSV isolate Y35. Experiments were performed with two independent T2 lines of TALE1 or TALE2 transgenic plants with each line of 10 plants. Before virus inoculation, the expression of TALE1 and TALE2 in all plants was determined by RT-PCR (Data not shown). After agrobacterium mediated inoculation, plants were maintained in a growth chamber for observation. Viral symptom development was monitored for a period of 30 days, and virus accumulation in the systemic leaves was detected using a Southern blot at 18 days post inoculation (dpi). Although both wild-type and transgenic plants developed typical leaf curl symptoms at 20 days post inoculation, viral symptoms on transgenic plants were significantly attenuated when compared to non-transgenic plants. For instance, wild-type plants displayed severe leaf curling symptoms, whereas only the leaf margins of transgenic plants showed curling (Figure 2A, bottom panel). Also, the height of transgenic plants was significantly greater than that of wild-type plants. Furthermore, while wild-type *N. benthamiana* plants had developed virus symptoms 9 dpi, the symptoms on both TALE1 and TALE2 transgenic plants did not appear until 14 dpi (Figure 2C). To further confirm these results, Southern blots were performed with a probe that amplified using primers Y35-F (5′-GATGCCTCAGCCAAGGAAAAC-3′) and Y35-R (5′-TCAACACGACGACGTCTG-3′) from the TbCSV genome and labeled with [α^−32^P] dCTP using the Random Primed Labeling System (Promega, Madison, WI) to evaluate TbCSV genomic DNA accumulation in wild-type and transgenic plants. Southern blot results showed that the levels of TbCSV genomic DNA accumulation in the upper leaves of both TALE1 and TALE2 transgenic plants were lower than that of wild-type plants (Figure 2D), suggesting that both TALE1 and TALE2 transgenic plants can inhibit TbCSV replication. Thus, these results indicate that transgenic plants expressing TALE1 or TALE2 confer partial resistance against TbCSV. We further challenged TALE1 and TALE2 transgenic plants with Tomato leaf curl Yunnan virus isolate Y194 (TLCYnV-Y194, accession number: HF674920), which shares 75.7% nucleotide sequence similarity with TbCSV. As shown in Supplementary Figure 2, the transgenic plants also showed partial resistance to TLCYnV-Y194 as indicated by viral symptoms and genomic DNA accumulation.

**Figure 3:**
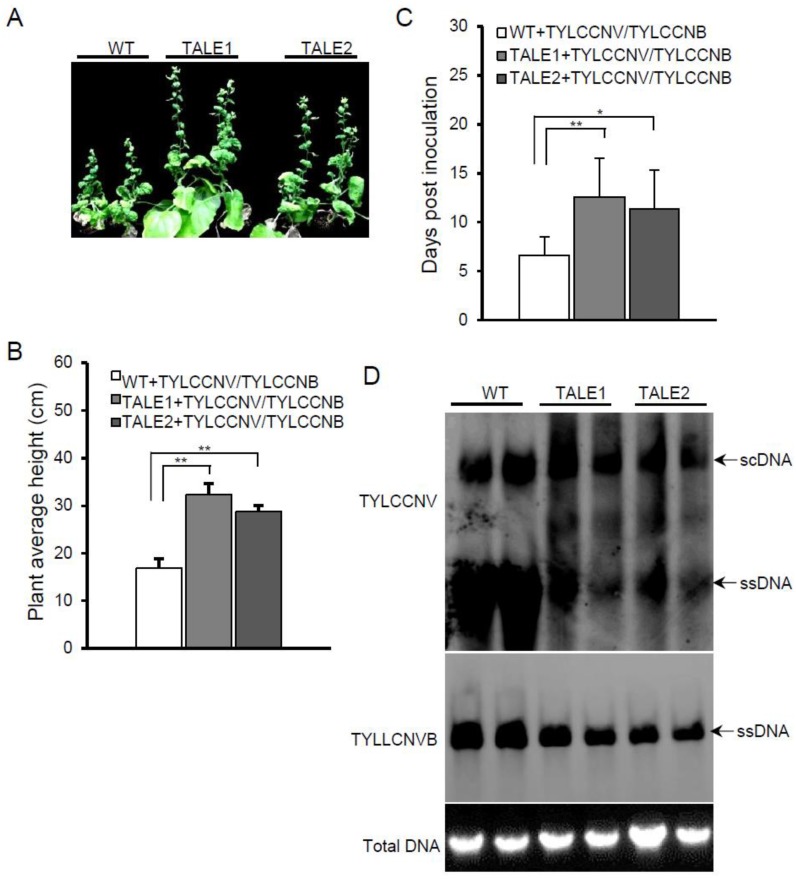
Transgenic plants show resistance to TYCCNV and its associated betasatellite (TYCCNB). (**A**) Symptoms induced by TYCCNV and TYCCNB on wild-type and TALE1 and TALE2 transgenic plants at 20 dpi; (**B**) Average plant height wild-type and transgenic TALE1 and TALE2 plants after infected by TYCCNV and TYCCNB. Numbers were calculated from two independent trials with each trial of 10 plants. ** indicates *p <* 0.01; (**C**) Average time for TYCCNV and TYCCNB inducing viral symptom on systemic leaves of wild-type and transgenic plants. Numbers were calculated from two independent trials with each trial of 10 plants. * indicates *p <* 0.05, whereas ** indicates *p <* 0.01; (**D**) Southern blot detection of TYCCNV (upper panel) and TYCCNB (middle panel) genomic DNA. An ethidium bromide stained gel was shown at the bottom for indicating DNA loadings.

Many monopartite begomoviruses are associated with satellite molecules, such as alphasatellite and betasatellite. Satellite DNAs are dependent on helper viruses for their replication, encapsidation, and transmission between hosts. However, satellites may benefit the helper virus in many aspects. For instance, the protein C1 encoded by betasatellite can suppress RNA silencing and increase replication of the helper virus [5]. To test whether transgenic plants can still provide resistance in the presence of betasatellites, we challenged TALE1 and TALE2 transgenic lines with Tomato yellow leaf curl China virus isolate Y10 (TYLCCNV, accession number: AJ319675) alone or together with its associated betasatellite (TYLCCNB, accession number: AJ421621). TYLCCNV shares 80.2% and 82.5% nucleotide sequence similarity with TbCSV and TLCYnV, respectively. As reported earlier [23], TYLCCNV, alone, did not induce visible viral symptoms on either wild-type or transgenic *N. benthamiana* plants (Supplementary Figure 3A). Interestingly, the transgenic plants infected with TYLCCNV were significantly taller than the virus-infected wild-type plants (Supplementary Figure 3B), suggesting that the infection of TYLCCNV actually affected *N. benthamiana* growth although it did not induce other obvious viral symptoms. Southern blot with a probe amplified with primers Y10A-F (5′-ATGTGGGATCCTCTGCTCAACGAGTTTC-3′) and Y10A-R (5′-CATCCTCAGACCTTGCGTTTCTTAAGAG-3′) showed that both TALE1 and TALE2 transgenic plants accumulated lower levels of TYLCCNV genomic DNA than wild-type plants (Supplementary Figure 3B) indicating that both TALE1 and TALE2 transgenic plants can also inhibit TYLCCNV replication. When plants were infected by TYLCCNV and its associated betasatellite (TYLCCNB), both wild-type and TALE1 and TALE2 transgenic plants developed typical begomovirus symptoms at 20 dpi (Figure 3A). However, the plant height of transgenic plants was significantly higher than that of wild-type plants when infected by TYLCCNV and TYLCCNB (Figure 3B). Furthermore, all wild-type plants showed viral symptoms at 9 dpi, whereas only about 30% of TALE1 and 40% of TALE2 transgenic plants showed viral symptoms at 9 dpi (data not shown). TYLCCNV and TYLCCNB took 6.6 ± 1.9 days to develop viral symptoms on wild-type plants, whereas it took 12.6 ± 3.9 and 11.4 ± 3.9 days to develop viral symptoms on TALE1 and TALE2 transgenic plants, respectively (Figure 3C). Southern blots showed that TALE1 and TALE2 inhibited not only the accumulation of TYLCCNV genomic DNA, but also the accumulation of TYLCCNB DNA (Figure 3D). These results suggest that transgenic plants expressing TALE1 or TALE2 confer partial viral resistance against TYLCCNV even in the presence of its betasatellite.

In this study, TALE technology was tested as a convenient protein platform for developing broad-spectrum resistance to begomoviruses. Two artificial TALE proteins were assembled based on two 12-nt conserved motifs in the genomes of begomoviruses. Transgenic *N. benthamiana* plants expressing these two artificial TALE proteins displayed certain a level of resistance to three begomoviruses, *i.e.*, delayed viral symptom development, attenuated viral symptoms, and reduced viral genomic DNA accumulation. Furthermore, the resistance was retained even in the presence of the associated virus betasetallite (Supplementary Figure 4). However, neither TALE1 nor TALE2 transgenic plants could completely inhibit virus replication, indicating that TALE’s ability to recognize a 12-nt target sequence may not be sufficient to completely inhibit virus replication. Another possibility is that the RVD (NK) we utilized in constructing our TALEs for recognizing G could lower the overall DNA-binding activity of the TALEs [22,24,25]. This issue has been solved by substitution of NK with the recently discovered RVD asparagine-histidine (NH) [24,25]. In the present study, the artificial TALE proteins do not contain any additionally functional domains, *i.e.*, the *Fok*I cleavage domain which is widely used for genome editing [19]. Therefore, TALE1 and TALE2 were supposed to function by directly binding to target sequences within challenging begomoviruses to prohibit their replication and/or affect the transcription of the genes they encoded. However, further experiments are needed to illustrate these possibilities. Nevertheless, our results show that TALE proteins are a very convenient platform for developing broad-spectrum resistance against begomoviruses. The TALE platform is ideal as it does not require a laborious and time-consuming screening process to obtain artificial TALE proteins with high binding specificity to target DNA. Furthermore, some naturally-occurring TALE repeats containing RVDs that can recognize more than one type of base, such as RVD asparagine-serine (NS) which recognizes A,C, G, or T, whereas NN recognizes A or G [15,16]. These repeats will allow for degenerative bases in target sequence, which should be especially useful when designing target sequences from a group of highly diverse viral sequences. In the future, we will further explore this possibility by optimizing target sequences through reduction of viral genome sequences to a certain geographic area, since most begomoviruses are not globally distributed and/or by adding additionally functional domains, such as nucleases, transcriptional activation or repression domains, and DNA methyltransferases.

## Supplementary Files

Supplementary File 1
