# Supplementary material for: Artificial TALE as a Convenient Protein Platform for Engineering Broad-Spectrum Resistance to Begomoviruses
Source: Viruses. 2015 Aug 20;7(8):4772–82. doi: 10.3390/v7082843 (PMC4576204; doi:10.3390/v7082843)
Supplement: Supplementary File 1 [file viruses-07-02843-s001.pdf]

Supplementary Materials

Supplementary Text 1:

Double-repeat units (underlines from 5’ to 3’ are restriction sites of Spe I, Nhe I, and Hind III, sequences with grey background encode RVDs):

dTpT

ACTAGTAATGGCGGCGGCAAGCAGGCGCTGGAGACGGTGCAGCGCCTGTTGCCGGGTCT
GTGCCAGGACCATGGCTTGTCGCCCGCCCAAGTAGTCGCGATTGCAAACAACGGGGGGG
GAAAACAAGCTCTCGAAATGGTTCAAAGATTGCTGCCCATGCTTTGTCAAGCGCACGGGC
TGACCCCGGACCAGGTGGTGGCCATCGCTAGCGGGAAGCTT

dTpC

ACTAGTAATGGCGGCGGCAAGCAGGCGCTGGAGACGGTGCAGCGCCTGTTGCCGGGTCT
GTGCCAGGACCATGGCTTGTCGCCCGCCCAAGTAGTCGCGATTGCAAACCACGACGGGGG
AAAACAAGCTCTCGAAATGGTTCAAAGATTGCTGCCCATGCTTTGTCAAGCGCACGGGCT
GACCCCGGACCAGGTGGTGGCCATCGCTAGCGGGAAGCTT

dTpA

ACTAGTAATGGCGGCGGCAAGCAGGCGCTGGAGACGGTGCAGCGCCTGTTGCCGGGTCT
GTGCCAGGACCATGGCTTGTCGCCCGCCCAAGTAGTCGCGATTGCAAACAACATTGGGGG
AAAACAAGCTCTCGAAATGGTTCAAAGATTGCTGCCCATGCTTTGTCAAGCGCACGGGCT
GACCCCGGACCAGGTGGTGGCCATCGCTAGCGGGAAGCTT

dTpG

ACTAGTAATGGCGGCGGCAAGCAGGCGCTGGAGACGGTGCAGCGCCTGTTGCCGGGTCT
GTGCCAGGACCATGGCTTGTCGCCCGCCCAAGTAGTCGCGATTGCAAACAACAAGGGGG
GAAAACAAGCTCTCGAAATGGTTCAAAGATTGCTGCCCATGCTTTGTCAAGCGCACGGGC
TGACCCCGGACCAGGTGGTGGCCATCGCTAGCGGGAAGCTT

dCpT

ACTAGTCACGATGGCGGCAAGCAGGCGCTGGAGACGGTGCAGCGCCTGTTGCCGGGTCTG
TGCCAGGACCATGGCTTGTCGCCCGCCCAAGTAGTCGCGATTGCAAACAACGGGGGGGG
AAAACAAGCTCTCGAAATGGTTCAAAGATTGCTGCCCATGCTTTGTCAAGCGCACGGGCT
GACCCCGGACCAGGTGGTGGCCATCGCTAGCGGGAAGCTT

dCpC

ACTAGTCACGATGGCGGCAAGCAGGCGCTGGAGACGGTGCAGCGCCTGTTGCCGGGTCTG
TGCCAGGACCATGGCTTGTCGCCCGCCCAAGTAGTCGCGATTGCAAACCATGACGGGGGA
AAACAAGCTCTCGAAATGGTTCAAAGATTGCTGCCCATGCTTTGTCAAGCGCACGGGCTG
ACCCCGGACCAGGTGGTGGCCATCGCTAGCGGGAAGCTT

dCpA

ACTAGTCACGATGGCGGCAAGCAGGCGCTGGAGACGGTGCAGCGCCTGTTGCCGGGTCTG
TGCCAGGACCATGGCTTGTCGCCCGCCCAAGTAGTCGCGATTGCAAACAACATTGGGGGA
AAACAAGCTCTCGAAATGGTTCAAAGATTGCTGCCCATGCTTTGTCAAGCGCACGGGCTG
ACCCCGGACCAGGTGGTGGCCATCGCTAGCGGGAAGCTT

dCpG

ACTAGTCACGATGGCGGCAAGCAGGCGCTGGAGACGGTGCAGCGCCTGTTGCCGGGTCTG
TGCCAGGACCATGGCTTGTCGCCCGCCCAAGTAGTCGCGATTGCAAACAACAAAGGGGG

AAAACAAGCTCTCGAAATGGTTCAAAGATTGCTGCCCATGCTTTGTCAAGCGCACGGGCT GACCCCGGACCAGGTGGTGGCCATCGCTAGCGGGAAGCTT

dApT

ACTAGTAATATTGGCGGCAAGCAGGCGCTGGAGACGGTGCAGCGCCTGTTGCCGGGTCTG TGCCAGGACCATGGCTTGTCGCCCGCCCAAGTAGTCGCGATTGCAAACAACGGGGGGGG AAAACAAGCTCTCGAAATGGTTCAAAGATTGCTGCCCATGCTTTGTCAAGCGCACGGGCT GACCCCGGACCAGGTGGTGGCCATCGCTAGCGGGAAGCTT

dApC

ACTAGTAATATTGGCGGCAAGCAGGCGCTGGAGACGGTGCAGCGCCTGTTGCCGGGTCTG TGCCAGGACCATGGCTTGTCGCCCGCCCAAGTAGTCGCGATTGCAAACCACGACGGGGGA AAACAAGCTCTCGAAATGGTTCAAAGATTGCTGCCCATGCTTTGTCAAGCGCACGGGCTG ACCCCGGACCAGGTGGTGGCCATCGCTAGCGGGAAGCTT

dApA

ACTAGTAATATTGGCGGCAAGCAGGCGCTGGAGACGGTGCAGCGCCTGTTGCCGGGTCTG TGCCAGGACCATGGCTTGTCGCCCGCCCAAGTAGTCGCGATTGCAAACAACATCGGGGGA AAACAAGCTCTCGAAATGGTTCAAAGATTGCTGCCCATGCTTTGTCAAGCGCACGGGCTG ACCCCGGACCAGGTGGTGGCCATCGCTAGCGGGAAGCTT

dApG

ACTAGTAATATTGGCGGCAAGCAGGCGCTGGAGACGGTGCAGCGCCTGTTGCCGGGTCTG TGCCAGGACCATGGCTTGTCGCCCGCCCAAGTAGTCGCGATTGCAAACAACAAAGGGGG AAAACAAGCTCTCGAAATGGTTCAAAGATTGCTGCCCATGCTTTGTCAAGCGCACGGGCT GACCCCGGACCAGGTGGTGGCCATCGCTAGCGGGAAGCTT

dGpT

ACTAGTAATAAGGGCGGCAAGCAGGCGCTGGAGACGGTGCAGCGCCTGTTGCCGGGTCT GTGCCAGGACCATGGCTTGTCGCCCGCCCAAGTAGTCGCGATTGCAAACAACGGGGGGG GAAAACAAGCTCTCGAAATGGTTCAAAGATTGCTGCCCATGCTTTGTCAAGCGCACGGGC TGACCCCGGACCAGGTGGTGGCCATCGCTAGCGGGAAGCTT

dGpC

ACTAGTAATAAGGGCGGCAAGCAGGCGCTGGAGACGGTGCAGCGCCTGTTGCCGGGTCT GTGCCAGGACCATGGCTTGTCGCCCGCCCAAGTAGTCGCGATTGCAAACCACGATGGGGG AAAACAAGCTCTCGAAATGGTTCAAAGATTGCTGCCCATGCTTTGTCAAGCGCACGGGCT GACCCCGGACCAGGTGGTGGCCATCGCTAGCGGGAAGCTT

dGpA

ACTAGTAATAAGGGCGGCAAGCAGGCGCTGGAGACGGTGCAGCGCCTGTTGCCGGGTCT GTGCCAGGACCATGGCTTGTCGCCCGCCCAAGTAGTCGCGATTGCAAACAACATCGGGGG AAAACAAGCTCTCGAAATGGTTCAAAGATTGCTGCCCATGCTTTGTCAAGCGCACGGGCT GACCCCGGACCAGGTGGTGGCCATCGCTAGCGGGAAGCTT

dGpG

ACTAGTAATAAGGGCGGCAAGCAGGCGCTGGAGACGGTGCAGCGCCTGTTGCCGGGTCT GTGCCAGGACCATGGCTTGTCGCCCGCCCAAGTAGTCGCGATTGCAAACAACAAAGGGG GAAAACAAGCTCTCGAAATGGTTCAAAGATTGCTGCCCATGCTTTGTCAAGCGCACGGGC TGACCCCGGACCAGGTGGTGGCCATCGCTAGCGGGAAGCTT

Flank_seq1 (underlines are *Nde* I, *Nhe* I, *Hind* III, and *Xho* I restriction sites):
CCATATGGTGGATCTACGCACGCTCGGCTACAGCCAGCAGCAACAGGAGAAGATCAAAC
CGAAGGTTCGTTCGACAGTGGCGCAGCACCACGAGGCACTGGTCGGCCATGGGTTTACAC
ACGCGCACATCGTTGCGCTCAGCCAACACCCGGCAGCGTTAGGGACCGTCGCTGTCAAGT
ATCAGGACATGATCGCAGCGTTGCCAGAGGCGACACACGAAGCGATCGTTGGCGTCGGC
AAACAGTGGTCCGGCGCACGCGCTCTGGAGGCCTTGCTCACGGTGGCGGGAGAGTTGAG
AGGTCCACCGTTACAGTTGGACACAGGCCAACTTCTCAAGATTGCAAAACGTGGCGGCGT
GACCGCAGTGGAGGCAGTGCATGCATGGCGCAATGCACTGACGGGTGCCCCCCTGAACCT
GACCCCGGACCAGGTGGTGGCCATCGCTAGCAATGGCGGCGGCAAGCAGGCGCTGGAGA
GCATTTTTGCACAGTTATCTCGCCCTGATCCGGCGTTGGCCGCGTTGACCAACGACCGCCT
CGTCGCCTTGGCCTGCATCGGCGGACGCTCTGCGCTGAATGCAGTGAAAGACGGATTGCC
GAATGCGCTGACATTGATCAGAAGAGCCAATAGCCGTATTCCCGAACGCACAAAGCTTGG
GCTCGAGG

TALE1 (underlines are *Nde* I, *Hind* III, and *Xho* I restriction sites):
CCATATGGTGGATCTACGCACGCTCGGCTACAGCCAGCAGCAACAGGAGAAGATCAAAC
CGAAGGTTCGTTCGACAGTGGCGCAGCACCACGAGGCACTGGTCGGCCATGGGTTTACAC
ACGCGCACATCGTTGCGCTCAGCCAACACCCGGCAGCGTTAGGGACCGTCGCTGTCAAGT
ATCAGGACATGATCGCAGCGTTGCCAGAGGCGACACACGAAGCGATCGTTGGCGTCGGC
AAACAGTGGTCCGGCGCACGCGCTCTGGAGGCCTTGCTCACGGTGGCGGGAGAGTTGAG
AGGTCCACCGTTACAGTTGGACACAGGCCAACTTCTCAAGATTGCAAAACGTGGCGGCGT
GACCGCAGTGGAGGCAGTGCATGCATGGCGCAATGCACTGACGGGTGCCCCCCTGAACCT
GACCCCGGACCAGGTGGTGGCCATCGCTAGTAATATTGGCGGCAAGCAGGCGCTGGAGA
CGGTGCAGCGCCTGTTGCCGGGTCTGTGCCAGGACCATGGCTTGTCGCCCGCCCAAGTAG
TCGCGATTGCAAACCACGACGGGGGAAAACAAGCTCTCGAAATGGTTCAAAGATTGCTGC
CCATGCTTTGTCAAGCGCACGGGCTGACCCCGGACCAGGTGGTGGCCATCGCTAGTAATA
AGGGCGGCAAGCAGGCGCTGGAGACGGTGCAGCGCCTGTTGCCGGGTCTGTGCCAGGAC
CATGGCTTGTCGCCCGCCCAAGTAGTCGCGATTGCAAACAACAAAGGGGGAAAACAAGC
TCTCGAAATGGTTCAAAGATTGCTGCCCATGCTTTGTCAAGCGCACGGGCTGACCCCGGA
CCAGGTGGTGGCCATCGCTAGTAATATTGGCGGCAAGCAGGCGCTGGAGACGGTGCAGC
GCCTGTTGCCGGGTCTGTGCCAGGACCATGGCTTGTCGCCCGCCCAAGTAGTCGCGATTG
CAAACAACGGGGGGGGAAAACAAGCTCTCGAAATGGTTCAAAGATTGCTGCCCATGCTTT
GTCAAGCGCACGGGCTGACCCCGGACCAGGTGGTGGCCATCGCTAGTAATAAGGGCGGC
AAGCAGGCGCTGGAGACGGTGCAGCGCCTGTTGCCGGGTCTGTGCCAGGACCATGGCTTG
TCGCCCGCCCAAGTAGTCGCGATTGCAAACAACAAAGGGGGAAAACAAGCTCTCGAAAT
GGTTCAAAGATTGCTGCCCATGCTTTGTCAAGCGCACGGGCTGACCCCGGACCAGGTGGT
GGCCATCGCTAGTCACGATGGCGGCAAGCAGGCGCTGGAGACGGTGCAGCGCCTGTTGCC
GGGTCTGTGCCAGGACCATGGCTTGTCGCCCGCCCAAGTAGTCGCGATTGCAAACCATGA
CGGGGGAAAACAAGCTCTCGAAATGGTTCAAAGATTGCTGCCCATGCTTTGTCAAGCGCA
CGGGCTGACCCCGGACCAGGTGGTGGCCATCGCTAGTAATAAGGGCGGCAAGCAGGCGC
TGGAGACGGTGCAGCGCCTGTTGCCGGGTCTGTGCCAGGACCATGGCTTGTCGCCCGCCC
AAGTAGTCGCGATTGCAAACCACGATGGGGGAAAACAAGCTCTCGAAATGGTTCAAAGA

TTGCTGCCCATGCTTTGTCAAGCGCACGGGCTGACCCCGGACCAGGTGGTGGCCATCGCT
AGCAATGGCGGCGGCAAGCAGGCGCTGGAGAGCATTTTTGCACAGTTATCTCGCCCTGAT
CCGGCGTTGGCCGCGTTGACCAACGACCGCCTCGTCGCCTTGGCCTGCATCGGCGGACGC
TCTGCGCTGAATGCAGTGAAAGACGGATTGCCGAATGCGCTGACATTGATCAGAAGAGCC
AATAGCCGTATTCCCGAACGCACAAAGCTTGGGCTCGAGG

TALE2(underlines are Nde I, Hind III, and Xho I restriction sites):
CCATATGGTGGATCTACGCACGCTCGGCTACAGCCAGCAGCAACAGGAGAAGATCAAAC
CGAAGGTTCGTTCGACAGTGGCGCAGCACCACGAGGCACTGGTCGGCCATGGGTTTACAC
ACGCGCACATCGTTGCGCTCAGCCAACACCCGGCAGCGTTAGGGACCGTCGCTGTCAAGT
ATCAGGACATGATCGCAGCGTTGCCAGAGGCGACACACGAAGCGATCGTTGGCGTCGGC
AAACAGTGGTCCGGCGCACGCGCTCTGGAGGCCTTGCTCACGGTGGCGGGAGAGTTGAG
AGGTCCACCGTTACAGTTGGACACAGGCCAACTTCTCAAGATTGCAAAACGTGGCGGCGT
GACCGCAGTGGAGGCAGTGCATGCATGGCGCAATGCACTGACGGGTGCCCCCCTGAACCT
GACCCCGGACCAGGTGGTGGCCATCGCTAGTAATAAGGGCGGCAAGCAGGCGCTGGAGA
CGGTGCAGCGCCTGTTGCCGGGTCTGTGCCAGGACCATGGCTTGTCGCCCGCCCAAGTAG
TCGCGATTGCAAACAACAAAGGGGGAAAACAAGCTCTCGAAATGGTTCAAAGATTGCTG
CCCATGCTTTGTCAAGCGCACGGGCTGACCCCGGACCAGGTGGTGGCCATCGCTAGTCAC
GATGGCGGCAAGCAGGCGCTGGAGACGGTGCAGCGCCTGTTGCCGGGTCTGTGCCAGGA
CCATGGCTTGTCGCCCGCCCAAGTAGTCGCGATTGCAAACCATGACGGGGGAAAACAAGC
TCTCGAAATGGTTCAAAGATTGCTGCCCATGCTTTGTCAAGCGCACGGGCTGACCCCGGA
CCAGGTGGTGGCCATCGCTAGTAATAAGGGCGGCAAGCAGGCGCTGGAGACGGTGCAGC
GCCTGTTGCCGGGTCTGTGCCAGGACCATGGCTTGTCGCCCGCCCAAGTAGTCGCGATTG
CAAACCACGATGGGGGAAAACAAGCTCTCGAAATGGTTCAAAGATTGCTGCCCATGCTTT
GTCAAGCGCACGGGCTGACCCCGGACCAGGTGGTGGCCATCGCTAGTAATAAGGGCGGC
AAGCAGGCGCTGGAGACGGTGCAGCGCCTGTTGCCGGGTCTGTGCCAGGACCATGGCTTG
TCGCCCGCCCAAGTAGTCGCGATTGCAAACCACGATGGGGGAAAACAAGCTCTCGAAAT
GGTTCAAAGATTGCTGCCCATGCTTTGTCAAGCGCACGGGCTGACCCCGGACCAGGTGGT
GGCCATCGCTAGTAATATTGGCGGCAAGCAGGCGCTGGAGACGGTGCAGCGCCTGTTGCC
GGGTCTGTGCCAGGACCATGGCTTGTCGCCCGCCCAAGTAGTCGCGATTGCAAACAACAA
AGGGGGAAAACAAGCTCTCGAAATGGTTCAAAGATTGCTGCCCATGCTTTGTCAAGCGCA
CGGGCTGACCCCGGACCAGGTGGTGGCCATCGCTAGTCACGATGGCGGCAAGCAGGCGC
TGGAGACGGTGCAGCGCCTGTTGCCGGGTCTGTGCCAGGACCATGGCTTGTCGCCCGCCC
AAGTAGTCGCGATTGCAAACAACAAAGGGGGAAAACAAGCTCTCGAAATGGTTCAAAGA
TTGCTGCCCATGCTTTGTCAAGCGCACGGGCTGACCCCGGACCAGGTGGTGGCCATCGCT
AGCAATGGCGGCGGCAAGCAGGCGCTGGAGAGCATTTTTGCACAGTTATCTCGCCCTGAT
CCGGCGTTGGCCGCGTTGACCAACGACCGCCTCGTCGCCTTGGCCTGCATCGGCGGACGC
TCTGCGCTGAATGCAGTGAAAGACGGATTGCCGAATGCGCTGACATTGATCAGAAGAGCC
AATAGCCGTATTCCCGAACGCACAAAGCTTGGGCTCGAGG

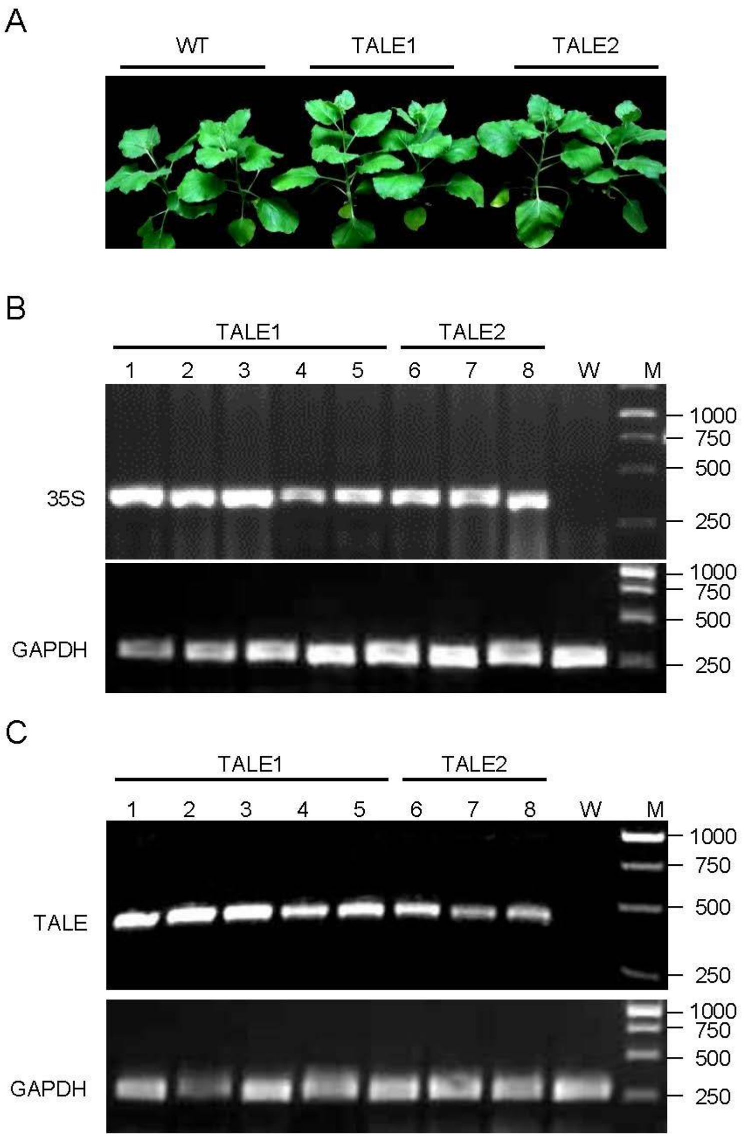

Figure S1. Production and analysis transgenic *N. benthamiana* plants. (**A**) Phenotypes of TALE1 and TALE2 transgenic *N. benthamiana* plants. Pictures were taken at 4 weeks post germination; (**B**) Detection of T-DNA insertion in transgenic *N. benthamiana* plants. The presence of T-DNA insertion in *N. benthamiana* genome was determined by amplified 350 bp fragments from 35S promoter. A 250 bp fragment of GAPDH gene was amplified as loading controls; (**C**) Detection of expression of TALE1 and TALE2 in transgenic *N. benthamiana* plants. A 420 bp fragment of artificial *TALE1* and *TALE2* was amplified from cDNA transcribed from total RNA extracted from transgenic *N. benthamiana* leaves with oligodT$_{15}$. A 250 bp fragment of GAPDH gene was amplified as loading controls. DNA ladders from top to bottoms represent 1000, 750, 500, and 250 bp binds, respectively.

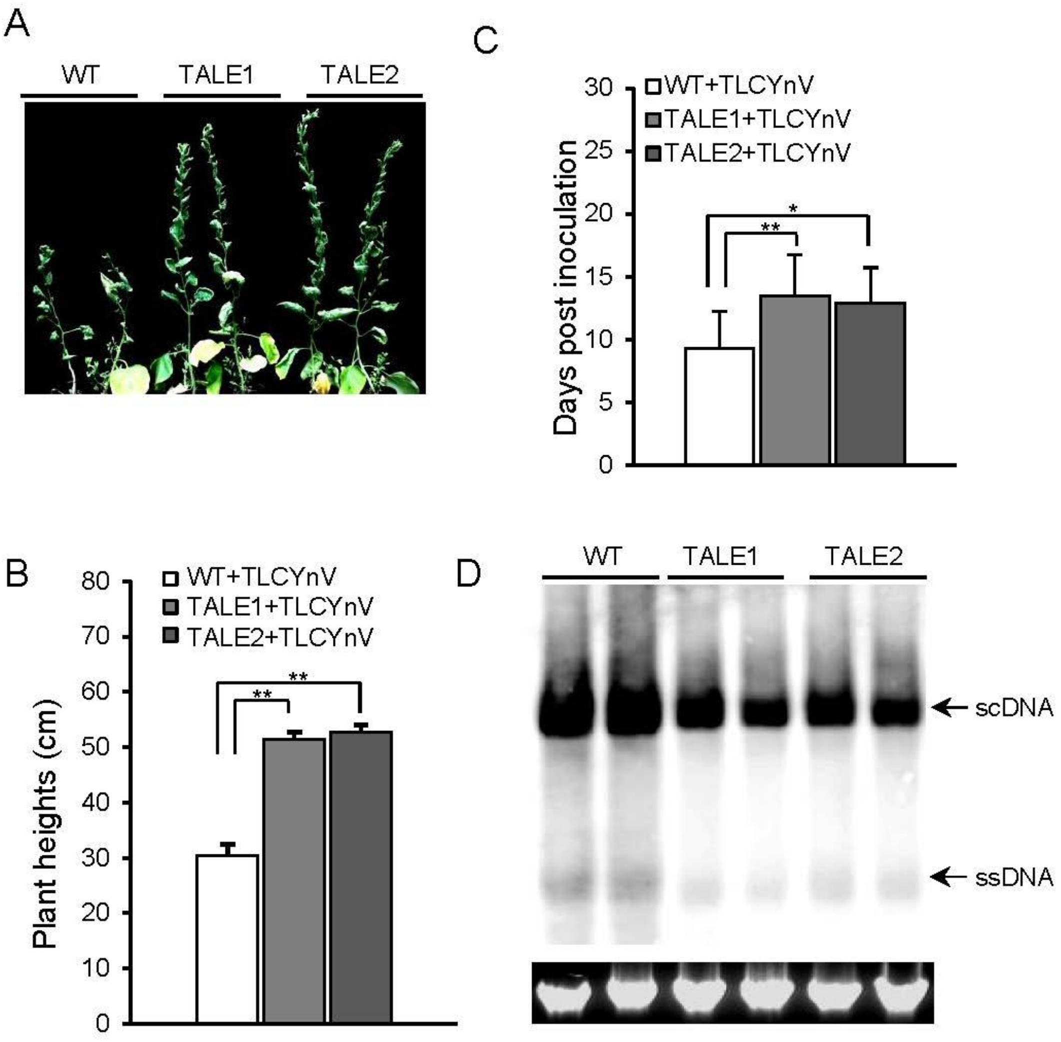

Figure S2. Transgenic plants show resistance to TLCYnV. (**A**) Symptoms induced by TLCYnV on wild-type and transgenic TALE1 and TALE2 *N. benthamiana* plants. Picture was taken at 20 dpi; (**B**) Average plant height of wild-type and transgenic TALE1, and TALE2 plants infected by TLCYnV. Numbers were calculated from two independent trials with each trial of 10 plants. ** indicates $P< 0.01$; (**C**) Average time for TLCYnV inducing viral symptom on systemic leaves of wild-type and transgenic plants. Numbers were calculated from two independent trials with each trial of 10 plants. * indicates $P < 0.05$, whereas ** indicates $P < 0.01$; (**D**) Southern blot detection of TLCYnV genomic DNA in wild-type, TALE1, and TALE2 transgenic plants. An ethidium bromide stained gel was shown at the bottom for indicating DNA loadings.

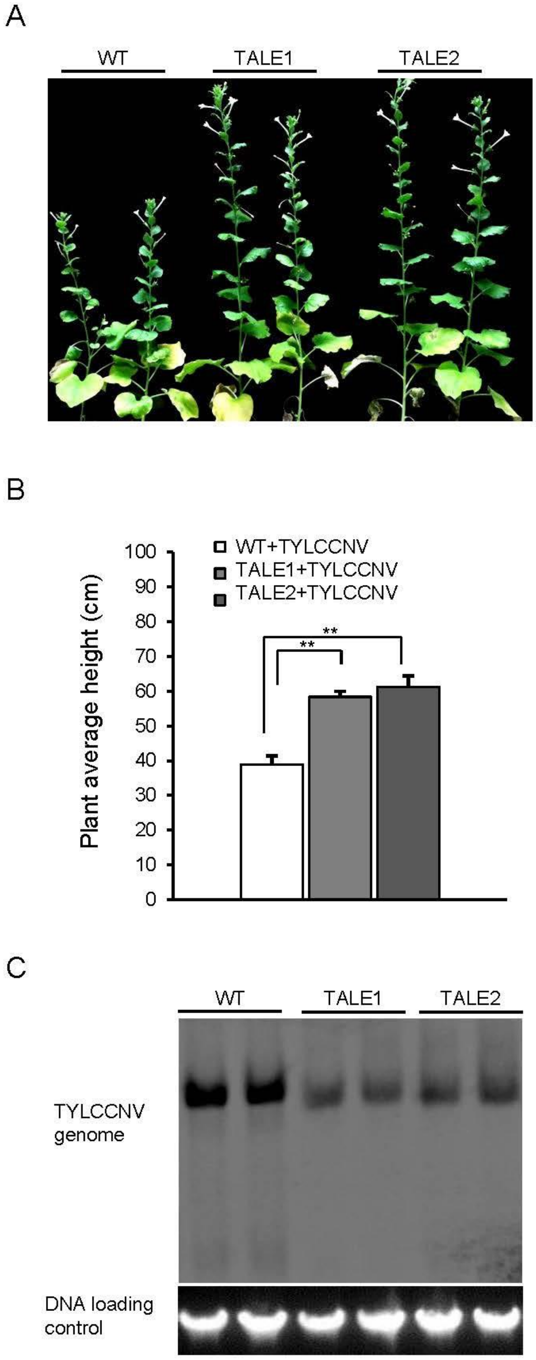

Figure S3. Transgenic plants show resistance to TYCCNV. (**A**) Phenotypes of wild-type and transgenic TALE1 and TALE2 *N. benthamiana* plants infected by TYCCNV at 20 dpi; (**B**) Average plant height of wild-type and transgenic TALE1 and TALE2 plants after infecting by TYCCNV. Numbers were calculated from two independent trials with each trial of 10 plants. ** indicates $P < 0.01$; (**C**) Southern blot detection of TYCCNV genomic DNA in wild-type, TALE1, and TALE2 transgenic plants. An ethidium bromide stained gel showed at the bottom for indicating DNA loadings.

© 2015 by the authors; licensee MDPI, Basel, Switzerland. This article is an open access article distributed under the terms and conditions of the Creative Commons Attribution license (http://creativecommons.org/licenses/by/4.0/).
